# 4-Oxo-1,4-dihydro­benzo[*h*][1,3]thia­zeto[3,2-*a*]quinoline-1,3-dicarb­oxy­lic acid

**DOI:** 10.1107/S1600536811003333

**Published:** 2011-01-29

**Authors:** Louise N. Dawe, Abeer Ahmed, Mohsen Daneshtalab

**Affiliations:** a Department of Chemistry, Memorial University of Newfoundland, St John’s, NL, Canada A1B 3X7; b School of Pharmacy, Memorial University of Newfoundland, St John’s, NL, Canada A1B 3V6

## Abstract

In the title mol­ecule, C_16_H_9_NO_5_S, there is an intra­molecular O—H⋯O hydrogen bond involving the quinolone carbonyl O atom and a carboxyl OH group. In the crystal, inter­molecular O—H⋯O hydrogen bonds between the carbonyl group of the quinolone carboxyl group, and a second carboxyl group on the thia­zeto moiety lead to the formation of chains propagating along [201] and perpendicular to the π-stacks of mol­ecules.

## Related literature

For background to the biological importance of thia­zetoquinoline anti­biotics, see: Ozaki *et al.* (1991 ▶). For similar work using different procedures, see: Ito *et al.* (1992 ▶, 1994 ▶); Matsuoka *et al.* (1999 ▶).
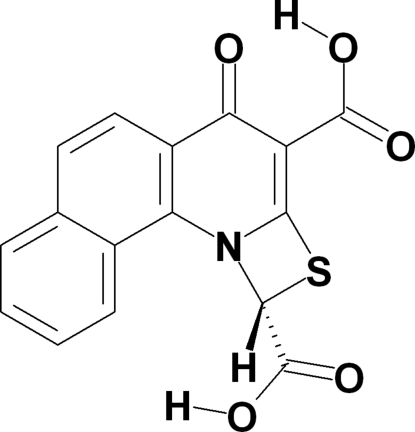


## Experimental

### 

#### Crystal data

C_16_H_9_NO_5_S
*M* *_r_* = 327.31
Monoclinic, 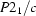

*a* = 7.237 (2) Å
*b* = 16.171 (5) Å
*c* = 11.929 (4) Å
β = 106.081 (8)°
*V* = 1341.5 (7) Å^3^
*Z* = 4
Mo *K*α radiation
μ = 0.27 mm^−1^
*T* = 153 K
0.18 × 0.04 × 0.04 mm

#### Data collection

Rigaku Saturn diffractometer
Absorption correction: numerical (*ABSCOR*; Higashi, 1999 ▶) *T*_min_ = 0.974, *T*_max_ = 0.996
17300 measured reflections
2769 independent reflections
2614 reflections with *I* > 2σ(*I*)
*R*_int_ = 0.074

#### Refinement

*R*[*F* ^2^ > 2σ(*F* ^2^)] = 0.088
*wR*(*F* ^2^) = 0.164
*S* = 1.30
2769 reflections
214 parameters
2 restraints
H atoms treated by a mixture of independent and constrained refinement
Δρ_max_ = 0.31 e Å^−3^
Δρ_min_ = −0.31 e Å^−3^

### 

Data collection: *CrystalClear* (Rigaku, 2005 ▶); cell refinement: *CrystalClear*; data reduction: *CrystalClear*; program(s) used to solve structure: *SHELXS97* (Sheldrick, 2008 ▶); program(s) used to refine structure: *SHELXL97* (Sheldrick, 2008 ▶); molecular graphics: *Mercury* (Macrae *et al.*, 2006 ▶); software used to prepare material for publication: *publCIF* (Westrip, 2010 ▶).

## Supplementary Material

Crystal structure: contains datablocks I, global. DOI: 10.1107/S1600536811003333/su2249sup1.cif

Structure factors: contains datablocks I. DOI: 10.1107/S1600536811003333/su2249Isup2.hkl

Additional supplementary materials:  crystallographic information; 3D view; checkCIF report

## Figures and Tables

**Table 1:** Hydrogen-bond geometry (Å, °)

*D*—H⋯*A*	*D*—H	H⋯*A*	*D*⋯*A*	*D*—H⋯*A*
O5—H5*A*⋯O1	0.96 (4)	1.57 (4)	2.504 (4)	161 (4)
O3—H3⋯O4^i^	0.97 (3)	1.62 (3)	2.569 (4)	166 (3)

Symmetry code: (i) 
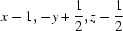
.

**Table 2:** π⋯π inter­actions (Å, °) Angle of elevation defined as the angle of the *Cg*(*I*)→*Cg*(*J*) vector and the normal to plane *J. Cg*1, *Cg*2 and *Cg*3 are the centroids of the C7–C12, N1/C1–C4/C13 and C4–C7/C12/C13 rings, respectively.

π⋯π	Distance	Angle of Elevation
*Cg*1⋯*Cg*2^i^	3.560 (2)	19.56
*Cg*3⋯*Cg*2^i^	3.644 (2)	22.75
*Cg*3⋯*Cg*3^i^	3.688 (2)	24.39

Symmetry code: (i) −*x* + 1, −*y*, −*z*.

**Fig. 1.**
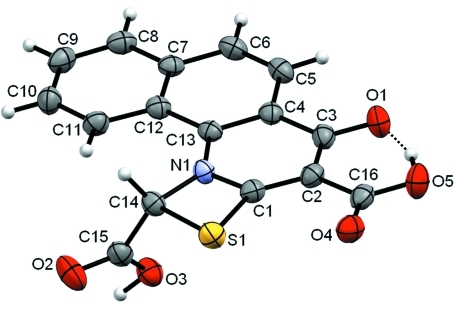
A view of the molecular structure of the title molecule, with displacement ellipsoids drawn at the 50% probability level.

**Fig. 2.**
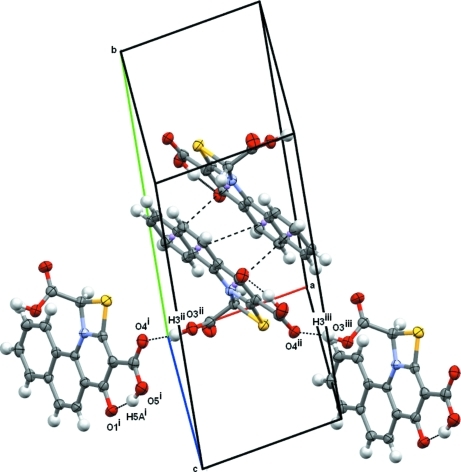
A partial view of the crystal packing of the title compound. Both the hydrogen bonding [symmetry codes: (i) x-1, y, z; (ii) x, -y+1/2, z+1/2; (iii) x+1, y, z+1] and π···π interactions [symmetry codes: (ii) x, -y+1/2, z+1/2; (iv) -x+1, y+1/2, -z+1/2] are shown as dashed lines; ring centroids are marked by small spheres. See Tables 1 and 2 for details.

## supplementary crystallographic information

### Comment

4-oxo-1,4-dihydroquinoline-3-carboxylic acid derivatives (quinolones) are an important class of antibacterial agents, and a significant market exists for thiazetoquinoline antibiotics (Matsuoka *et al.*,1999; Ito *et al.*, 1992; Ito *et al.*, 1994; Ozaki *et al.*, 1991). To this end, the title comound was obtained from the reaction of ethyl 2-{[2- ethoxy-2-oxoethyl)thio)-4-hydroxybenzo[*h*]quinoline-3-carboxylate with 1,2-dibromopropane in the presence of a catalytic amount of KI, followed by saponification using sodium hydroxide.

The molecular structure of the title molecule is shown in Fig. 1. It exhibits intra- (O5—H5a···O1) and intermolecular (O3—H3···O4^i^) hydrogen bonding (Table 1 and Fig. 2) leading to a chain-like arrangement of molecules which run along [201] and perpendicular to the π stacks (Fig. 2). Centroid-centroid distances range from 3.560 (2) to 3.688 (2) Å with angles of elevation between 19.56 and 24.39° (Table 2), while the inter-planar distance, as defined by the adjacent 14-atom (N1,C1—C13) ring system is 3.34 (1) Å.

### Experimental

To a mixture of ethyl 2-{[2- ethoxy-2-oxoethyl)thio)-4-hydroxybenzo[*h*]quinoline-3-carboxylate (1 mmol) and K_2_CO_3_ (2.8 mmol) in dry DMF (25 ml) under a nitrogen atmosphere was added 1,2-dibromopropane (2.8 mmol) along with a catlytic amount of KI. The reaction mixture was heated at 343 K for 24 h, and then poured into ice-H_2_O. The resulting thiazetoquinoline derivative was collected by filtration. The separated product was reacted with sodium hydroxide (2.2 mmol) in water (20 ml) and heated at 373 K for 3–4 h. After being cooled, the reaction mixture was neutralized with hydrochloric acid (1 mol/*L*), extracted with CH_2_Cl_2_, dried over MgSO_4_, and then evaporated. The obtained solid was purified by recrystallization from ethanol to afford the title compound as a yellowish white powder. Mp. 508 K, yield = 39%. ^1^H-NMR and ^13^C-NMR data are given in the archived CIF.

### Refinement

The OH H-atoms, H3 and H5a, were located from difference Fourier maps, and were refined with distance restraints: O-H = 0.96 (3) Å, with *U*_iso_(H) = 1.2U_eq_(O). The C-bound H-atoms were included in calculated positions and treated as riding atoms: C-H = 0.95, 0.98, 0.99 and 1.0 Å for H-aromatic, H-methyl, H-methylene and methine H-atoms, respectively, with U_iso_(H) = k × U_eq_(parent C-atom), where k = 1.5 for H-methyl and k = 1.2 for all other H-atoms.

### Crystal data

**Table:**

C_16_H_9_NO_5_S	*F*(000) = 672
*M**_r_* = 327.31	*D*_x_ = 1.621 Mg m^−^^3^
Monoclinic, *P*2_1_/*c*	Mo *K*α radiation, λ = 0.71075 Å
Hall symbol: -P 2ybc	Cell parameters from 4915 reflections
*a* = 7.237 (2) Å	θ = 2.2–30.6°
*b* = 16.171 (5) Å	µ = 0.27 mm^−^^1^
*c* = 11.929 (4) Å	*T* = 153 K
β = 106.081 (8)°	Needle, colourless
*V* = 1341.5 (7) Å^3^	0.18 × 0.04 × 0.04 mm
*Z* = 4	

### Data collection

**Table:**

Rigaku Saturn diffractometer	2769 independent reflections
Radiation source: fine-focus sealed tube	2614 reflections with *I* > 2σ(*I*)
graphite - Rigaku SHINE	*R*_int_ = 0.074
Detector resolution: 14.63 pixels mm^-1^	θ_max_ = 26.5°, θ_min_ = 2.5°
ω scans	*h* = −9→9
Absorption correction: numerical (*ABSCOR*; Higashi, 1999)	*k* = −20→20
*T*_min_ = 0.974, *T*_max_ = 0.996	*l* = −14→14
17300 measured reflections	

### Refinement

**Table:**

Refinement on *F*^2^	Primary atom site location: structure-invariant direct methods
Least-squares matrix: full	Secondary atom site location: difference Fourier map
*R*[*F*^2^ > 2σ(*F*^2^)] = 0.088	Hydrogen site location: inferred from neighbouring sites
*wR*(*F*^2^) = 0.164	H atoms treated by a mixture of independent and constrained refinement
*S* = 1.30	*w* = 1/[σ^2^(*F*_o_^2^) + (0.0366*P*)^2^ + 2.1946*P*] where *P* = (*F*_o_^2^ + 2*F*_c_^2^)/3
2769 reflections	(Δ/σ)_max_ < 0.001
214 parameters	Δρ_max_ = 0.31 e Å^−^^3^
2 restraints	Δρ_min_ = −0.31 e Å^−^^3^

### Special details

**Table:**

Experimental. Spectroscopic data:^1^H-NMR: (500 MHz, DMSO-d6): δ= 8.27(1*H*, d, *J*=8.8), 8.25(1*H*, d, *J*=8.4), 8.17(1*H*, d, *J*=7.5), 8.02(1*H*, d, *J*=8.80), 7.83(1*H*, dd, *J*=11.0, 4.0), 7.81–7.76(1*H*, m), 7.73(1*H*, s)}. ^13^C-NMR: (500 MHz, DMSO-d6): δ= 175.76, 165.64, 165.25, 164.26, 136.09, 135.26, 129.58, 128.97, 127.58, 126.05, 122.67, 122.33, 121.53, 121.15, 103.64, 70.43.
Geometry. All e.s.d.'s (except the e.s.d. in the dihedral angle between two l.s. planes) are estimated using the full covariance matrix. The cell e.s.d.'s are taken into account individually in the estimation of e.s.d.'s in distances, angles and torsion angles; correlations between e.s.d.'s in cell parameters are only used when they are defined by crystal symmetry. An approximate (isotropic) treatment of cell e.s.d.'s is used for estimating e.s.d.'s involving l.s. planes.
Refinement. Refinement of *F*^2^ against ALL reflections. The weighted *R*-factor *wR* and goodness of fit *S* are based on *F*^2^, conventional *R*-factors *R* are based on *F*, with *F* set to zero for negative *F*^2^. The threshold expression of *F*^2^ > σ(*F*^2^) is used only for calculating *R*-factors(gt) *etc*. and is not relevant to the choice of reflections for refinement. *R*-factors based on *F*^2^ are statistically about twice as large as those based on *F*, and *R*- factors based on ALL data will be even larger.

### Fractional atomic coordinates and isotropic or equivalent isotropic displacement parameters (Å^2^)

**Table:**

	*x*	*y*	*z*	*U*_iso_*/*U*_eq_	
S1	0.60834 (14)	0.26465 (6)	0.14885 (8)	0.0347 (3)	
O1	0.5029 (4)	−0.01520 (17)	0.3384 (2)	0.0387 (7)	
O2	0.2537 (4)	0.33079 (19)	−0.0999 (3)	0.0512 (8)	
O3	0.1270 (4)	0.25356 (17)	0.0178 (2)	0.0370 (6)	
O4	0.7870 (4)	0.20609 (17)	0.3966 (2)	0.0379 (7)	
O5	0.7226 (4)	0.08587 (19)	0.4695 (2)	0.0439 (7)	
N1	0.4277 (4)	0.14703 (17)	0.0711 (2)	0.0257 (6)	
C1	0.5397 (5)	0.1656 (2)	0.1790 (3)	0.0274 (7)	
C2	0.5731 (5)	0.1139 (2)	0.2722 (3)	0.0289 (8)	
C3	0.4829 (5)	0.0354 (2)	0.2544 (3)	0.0303 (8)	
C4	0.3696 (5)	0.0139 (2)	0.1369 (3)	0.0277 (8)	
C5	0.2892 (5)	−0.0668 (2)	0.1153 (3)	0.0318 (8)	
H5	0.3105	−0.1057	0.1774	0.038*	
C6	0.1828 (5)	−0.0887 (2)	0.0074 (3)	0.0317 (8)	
H6	0.1297	−0.1428	−0.0048	0.038*	
C7	0.1484 (5)	−0.0329 (2)	−0.0882 (3)	0.0281 (8)	
C8	0.0343 (5)	−0.0566 (2)	−0.2002 (3)	0.0327 (8)	
H8	−0.0180	−0.1108	−0.2118	0.039*	
C9	−0.0019 (5)	−0.0027 (3)	−0.2920 (3)	0.0364 (9)	
H9	−0.0819	−0.0190	−0.3661	0.044*	
C10	0.0790 (6)	0.0762 (2)	−0.2765 (3)	0.0359 (9)	
H10	0.0552	0.1130	−0.3410	0.043*	
C11	0.1927 (5)	0.1015 (2)	−0.1696 (3)	0.0324 (8)	
H11	0.2475	0.1553	−0.1611	0.039*	
C12	0.2288 (5)	0.0480 (2)	−0.0719 (3)	0.0275 (8)	
C13	0.3401 (5)	0.0702 (2)	0.0439 (3)	0.0254 (7)	
C14	0.4427 (5)	0.2249 (2)	0.0104 (3)	0.0298 (8)	
H14	0.5090	0.2174	−0.0521	0.036*	
C15	0.2618 (5)	0.2759 (2)	−0.0304 (3)	0.0327 (8)	
C16	0.7025 (5)	0.1379 (2)	0.3852 (3)	0.0332 (9)	
H5A	0.649 (6)	0.039 (2)	0.432 (4)	0.052*	
H3	0.008 (4)	0.277 (2)	−0.029 (3)	0.044*	

### Atomic displacement parameters (Å^2^)

**Table:**

	*U*^11^	*U*^22^	*U*^33^	*U*^12^	*U*^13^	*U*^23^
S1	0.0343 (5)	0.0303 (5)	0.0351 (5)	−0.0046 (4)	0.0023 (4)	−0.0016 (4)
O1	0.0403 (16)	0.0408 (16)	0.0313 (14)	0.0000 (12)	0.0036 (12)	0.0104 (12)
O2	0.0460 (17)	0.0450 (17)	0.062 (2)	0.0053 (14)	0.0138 (15)	0.0244 (16)
O3	0.0304 (14)	0.0391 (15)	0.0396 (15)	0.0034 (12)	0.0065 (12)	0.0059 (12)
O4	0.0335 (14)	0.0420 (16)	0.0337 (14)	−0.0013 (13)	0.0022 (11)	−0.0073 (12)
O5	0.0438 (17)	0.0558 (19)	0.0260 (14)	−0.0055 (14)	−0.0006 (12)	0.0038 (13)
N1	0.0257 (15)	0.0240 (15)	0.0263 (15)	−0.0017 (12)	0.0053 (12)	0.0024 (12)
C1	0.0220 (17)	0.0300 (18)	0.0289 (18)	−0.0005 (14)	0.0051 (14)	−0.0049 (15)
C2	0.0265 (18)	0.033 (2)	0.0273 (18)	−0.0005 (15)	0.0080 (14)	−0.0024 (15)
C3	0.0285 (18)	0.036 (2)	0.0268 (18)	0.0060 (16)	0.0076 (14)	0.0044 (15)
C4	0.0243 (17)	0.0300 (19)	0.0294 (18)	0.0035 (15)	0.0087 (14)	0.0009 (15)
C5	0.0285 (19)	0.0283 (19)	0.040 (2)	0.0039 (15)	0.0111 (16)	0.0066 (16)
C6	0.0278 (18)	0.0252 (19)	0.042 (2)	0.0007 (15)	0.0092 (16)	0.0008 (16)
C7	0.0237 (17)	0.0275 (18)	0.0326 (19)	0.0035 (14)	0.0072 (14)	−0.0029 (15)
C8	0.0259 (18)	0.033 (2)	0.039 (2)	−0.0002 (15)	0.0076 (16)	−0.0087 (17)
C9	0.0282 (19)	0.045 (2)	0.031 (2)	0.0022 (17)	0.0004 (15)	−0.0106 (17)
C10	0.039 (2)	0.038 (2)	0.0280 (19)	0.0004 (18)	0.0058 (16)	0.0025 (17)
C11	0.034 (2)	0.0300 (19)	0.0323 (19)	−0.0032 (16)	0.0082 (16)	−0.0041 (16)
C12	0.0220 (17)	0.0298 (19)	0.0303 (18)	0.0010 (14)	0.0065 (14)	−0.0007 (15)
C13	0.0224 (16)	0.0250 (17)	0.0302 (18)	0.0013 (14)	0.0094 (14)	0.0003 (15)
C14	0.0265 (18)	0.0290 (19)	0.0329 (19)	0.0002 (15)	0.0065 (15)	0.0027 (15)
C15	0.035 (2)	0.0271 (19)	0.0325 (19)	−0.0016 (16)	0.0029 (16)	−0.0013 (16)
C16	0.0300 (19)	0.044 (2)	0.0267 (19)	0.0051 (17)	0.0093 (15)	−0.0021 (17)

### Geometric parameters (Å, °)

**Table:**

S1—C1	1.744 (4)	C5—C6	1.351 (5)
S1—C14	1.866 (4)	C5—H5	0.9500
O1—C3	1.271 (4)	C6—C7	1.421 (5)
O2—C15	1.205 (4)	C6—H6	0.9500
O3—C15	1.314 (5)	C7—C8	1.416 (5)
O3—H3	0.963 (19)	C7—C12	1.423 (5)
O4—C16	1.250 (5)	C8—C9	1.366 (5)
O5—C16	1.288 (5)	C8—H8	0.9500
O5—H5A	0.965 (19)	C9—C10	1.394 (6)
N1—C1	1.350 (4)	C9—H9	0.9500
N1—C13	1.392 (4)	C10—C11	1.375 (5)
N1—C14	1.472 (4)	C10—H10	0.9500
C1—C2	1.358 (5)	C11—C12	1.416 (5)
C2—C3	1.416 (5)	C11—H11	0.9500
C2—C16	1.464 (5)	C12—C13	1.438 (5)
C3—C4	1.456 (5)	C14—C15	1.509 (5)
C4—C13	1.406 (5)	C14—H14	1.0000
C4—C5	1.423 (5)		
			
C1—S1—C14	73.49 (16)	C7—C8—H8	119.5
C15—O3—H3	107 (3)	C8—C9—C10	119.8 (3)
C16—O5—H5A	103 (3)	C8—C9—H9	120.1
C1—N1—C13	122.3 (3)	C10—C9—H9	120.1
C1—N1—C14	99.9 (3)	C11—C10—C9	121.1 (4)
C13—N1—C14	137.7 (3)	C11—C10—H10	119.5
N1—C1—C2	124.6 (3)	C9—C10—H10	119.5
N1—C1—S1	97.9 (2)	C10—C11—C12	120.5 (3)
C2—C1—S1	137.5 (3)	C10—C11—H11	119.7
C1—C2—C3	117.2 (3)	C12—C11—H11	119.7
C1—C2—C16	121.0 (3)	C11—C12—C7	118.3 (3)
C3—C2—C16	121.8 (3)	C11—C12—C13	124.3 (3)
O1—C3—C2	120.8 (3)	C7—C12—C13	117.3 (3)
O1—C3—C4	121.0 (3)	N1—C13—C4	115.7 (3)
C2—C3—C4	118.2 (3)	N1—C13—C12	123.0 (3)
C13—C4—C5	119.1 (3)	C4—C13—C12	121.3 (3)
C13—C4—C3	121.8 (3)	N1—C14—C15	116.8 (3)
C5—C4—C3	119.1 (3)	N1—C14—S1	88.6 (2)
C6—C5—C4	120.6 (3)	C15—C14—S1	112.6 (3)
C6—C5—H5	119.7	N1—C14—H14	112.3
C4—C5—H5	119.7	C15—C14—H14	112.3
C5—C6—C7	121.7 (3)	S1—C14—H14	112.3
C5—C6—H6	119.2	O2—C15—O3	127.1 (4)
C7—C6—H6	119.2	O2—C15—C14	119.8 (4)
C8—C7—C6	120.8 (3)	O3—C15—C14	113.1 (3)
C8—C7—C12	119.2 (3)	O4—C16—O5	123.0 (3)
C6—C7—C12	120.0 (3)	O4—C16—C2	120.2 (3)
C9—C8—C7	121.0 (4)	O5—C16—C2	116.8 (4)
C9—C8—H8	119.5		

### Hydrogen-bond geometry (Å, °)

**Table:**

*D*—H···*A*	*D*—H	H···*A*	*D*···*A*	*D*—H···*A*
O5—H5A···O1	0.96 (4)	1.57 (4)	2.504 (4)	161 (4)
O3—H3···O4^i^	0.97 (3)	1.62 (3)	2.569 (4)	166 (3)

Symmetry codes: (i) *x*−1, −*y*+1/2, *z*−1/2.

### Table 2 π···π interactions (Å, °)

Angle of elevation defined as the angle of the *Cg*(*I*)→*Cg*(*J*) vector and the normal to plane *J*. Cg1, Cg2 and Cg3 are the centroids of the C7–C12, N1/C1–C4/C13 and C4–C7/C12/C13 rings, respectively.

**Table:**

π···π	Distance	Angle of Elevation
Cg1···Cg2^i^	3.560 (2)	19.56
Cg3···Cg2^i^	3.644 (2)	22.75
Cg3···Cg3^i^	3.688 (2)	24.39

Symmetry code: (i) -x+1, -y, -z.
